# Artesunate–amodiaquine and artemether–lumefantrine for the treatment of uncomplicated falciparum malaria in Liberia: in vivo efficacy and frequency of molecular markers

**DOI:** 10.1186/s12936-022-04140-7

**Published:** 2022-04-27

**Authors:** Victor S. Koko, Marian Warsame, Benjamin Vonhm, Moses K. Jeuronlon, Didier Menard, Laurence Ma, Fahn Taweh, Lekilay Tehmeh, Paye Nyansaiye, Oliver J. Pratt, Sei Parwon, Patrick Kamara, Magnus Asinya, Aaron Kollie, Pascal Ringwald

**Affiliations:** 1 National Malaria Control Programme, Ministry of Health, Monrovia, Liberia; 2 School of Public Health and Community Medicine, University of Gothenburg, Gothenburg, Sweden; 3 National Public Health Institute of Liberia-NPHIL, Monrovia, Liberia; 4 World Health Organization, Country Office, Monrovia, Liberia; 5 Malaria Genetics and Resistance Unit, INSERM U1201, Institut Pasteur, Paris, France; 6 Laboratoire de Parasitologie et Mycologie Médicale, Hôpitaux Universitaires de Strasbourg, Strasbourg, France; 7 Institut de Parasitologie et Pathologie Tropicale, UR7292 Dynamique des Interactions Hôte Pathogène, Fédération de Médecine Translationnelle, Université de Strasbourg, Strasbourg, France; 8 Biomics Platform, C2RT, Institut Pasteur, Paris, France; 9 Quality Control Unit, Ministry of Health, Monrovia, Liberia; 10 Saclepea Comprehensive Health Center, Saclepea, Ministry of Health, Saclepea, Liberia; 11 Sinje Health Centre, Garwula, Ministery of Health, Garwula, Liberia; 12 Charles Henry Rennie Hospital, Kakata, Ministry of Health, Kakata, Liberia; 13 Bensonville Hospital, Bensonville, Ministry of Health, Bensonville, Liberia; 14 Global Malaria Programme, World Health Organization, Geneva, Switzerland

**Keywords:** Artesunate–amodiaquine, Artemether–lumefantrine, *Plasmodium falciparum*, Efficacy, Molecular markers of antimalarial drug resistance, Liberia

## Abstract

**Background:**

Artesunate–amodiaquine (ASAQ) and Artemether–lumefantrine (AL) are the recommended treatment for uncomplicated *Plasmodium falciparum* malaria in Liberia. Intermittent preventive treatment with sulfadoxine/pyrimethamine is also recommended for pregnant women. The therapeutic efficacy of Artesunate–amodiaquine and Artemether–lumefantrine, and the frequency of molecular markers associated with anti-malarial drug resistance were investigated.

**Methods:**

The therapeutic efficacy of ASAQ and AL was evaluated using the standard World Health Organization protocol (WHO. Methods for Surveillance of Antimalarial Drug Efficacy. Geneva: World Health Organization; 2009. https://www.who.int/malaria/publications/atoz/9789241597531/en/). Eligible children were recruited and monitored clinically and parasitologically for 28 days. Polymorphisms in the *Pfkelch 13*, chloroquine resistance transporter (*Pfcrt*), multidrug resistance 1 (*Pfmdr-1*), dihydrofolate reductase (*Pfdhfr*), and dihydropteroate synthase (*Pfdhps*) genes and copy number variations in the plasmepsin-2 (*Pfpm2)* gene were assessed in pretreatment samples.

**Results:**

Of the 359 children enrolled, 180 were treated with ASAQ (89 in Saclepea and 91 in Bensonville) and 179 with AL (90 in Sinje and 89 in Kakata). Of the recruited children, 332 (92.5%) reached study endpoints. PCR-corrected per-protocol analysis showed ACPR of 90.2% (95% CI: 78.6–96.7%) in Bensonville and 92.7% (95% CI: 83.4.8–96.5%) in Saclepea for ASAQ, while ACPR of 100% was observed in Kakata and Sinje for AL. In both treatment groups, only two patients had parasites on day 3. No artemisinin resistance associated *Pfkelch13* mutations or multiple copies of *Pfpm2* were found. Most samples tested had the *Pfcrt* 76 T mutation (80/91, 87.9%), while the *Pfmdr-1 86Y* (40/91, 44%) and 184F (47/91, 51.6%) mutations were less frequent. The *Pfdhfr* triple mutant (51I/59R/108 N) was the predominant allele (49.2%). For the *Pfdhps* gene, it was the 540E mutant (16.0%), and the 436A mutant (14.3%). The quintuple allele (51I/59R/108 N-437G/540E) was detected in only one isolate (1/357).

**Conclusion:**

This study reports a decline in the efficacy of ASAQ treatment, while AL remained highly effective, supporting the recent decision by NMCP to replace ASAQ with AL as first-line treatment for uncomplicated falciparum malaria. No association between the presence of the mutations in *Pfcrt* and *Pfmdr-1* and the risk of parasite recrudescence in patients treated with ASAQ was observed. Parasites with signatures known to be associated with artemisinin and piperaquine resistance were not detected. The very low frequency of the quintuple *Pfdhfr/Pfdhps* mutant haplotype supports the continued use of SP for IPTp. Monitoring of efficacy and resistance markers of routinely used anti-malarials is necessary to inform malaria treatment policy.

*Trial registration* ACTRN12617001064392.

**Supplementary Information:**

The online version contains supplementary material available at 10.1186/s12936-022-04140-7.

## Background

Malaria is a major health problem, with an estimated 241 million cases and 627,000 deaths worldwide, increasing from 227 million and 558,000 cases and deaths, respectively, in 2020 [1]. Countries in the World Health Organization (WHO) African Region accounted for 95% of the global malaria burden. Effective malaria treatment with artemisinin-based combination therapy is a critical component of recommended malaria interventions [2]. These anti-malarials combine potent and fast-acting artemisinin derivatives with long half-life partner drug, and include Artemether–lumefantrine (AL), Artesunate–amodiaquine (ASAQ), artesunate–mefloquine (ASMQ), dihydroartemisinin–piperaquine (DP), artesunate–sulfadoxine/pyrimethamine (ASSP) and artesunate–pyronaridine (ASPY) for the treatment of uncomplicated falciparum malaria. Recent studies have shown that ASAQ and AL, the most used artemisinin-based combinations, remain highly effective in Africa, achieving cure rates of > 90%, the recommended threshold for treatment policy change [3]. However, unacceptably high rates of treatment failure with ASSP in Somalia [4] and India [5] and DP in several Southeast Asian countries [6–8] have led to the abandonment of both first-line treatments in these countries. These findings are a reminder that anti-malarial drug resistance remains a serious threat to effective case management. Therefore, it is critical for malaria endemic countries to regularly monitor the efficacy of recommended artemisinin-based combinations to inform national treatment policy. Therapeutic efficacy study (TES), prospective evaluations of clinical and parasitological responses to treatment of uncomplicated malaria is the gold standard for generating evidence for national treatment guidelines [3]

In addition to TES, molecular markers (*i.e.,* genetic changes in *Plasmodium falciparum* genome), associated with anti-malarial drug resistance are complementary tools. Partial artemisinin resistance, defined as delayed parasite clearance following artesunate monotherapy or ACT [3], has emerged and spread in Southeast Asia [9]. Non-synonymous mutations in the propeller region of the *P. falciparum kelch13* (*Pfkelch13*) gene have been demonstrated to be a major determinant associated with artemisinin resistance [10, 11]. Since 2014, more than 260 non-synonymous *Pfkelch13* mutations have been detected worldwide, but only 21 have been validated (F446I, N458Y, M476I, Y493H, R539T, I543T, P553L, R561H, P574L, C580Y) or are suspected to be associated (P441L, G449A, C469F/Y, A481V, R515K, P527H, N537I/D, G538V, R622I, V568G, A675V) with partial artemisinin resistance [3]. The presence of *Pfkelch13* mutations known to be associated with artemisinin resistance in Africa has historically been rare and sporadic [3]. However, results of recent studies suggest the emergence and spread of indigenous *Pfkelch13* mutants (R561H in Rwanda and A675V and C469Y in Uganda) associated with delayed parasite clearance and in vitro artemisinin resistance [12–14].

High prevalence of multiple copies of the *plasmepsin 2* (*Pfpm2*) gene, a marker of piperaquine resistance [15], has been observed to be associated with DP treatment failure in Southeast Asian countries, where artemisinin resistance is frequent [16–18]. Studies on African isolates showed varying frequencies of multiple copies of *Pfpm2* gene: < 5% in Mozambique [19], 30.5% and 33.9% in Burkina Faso and Uganda, respectively [20], and up to 50% in Burundi [3]. However, in high transmission areas like Africa, the detection of minor clones with amplified *Pfpm2* in polyclonal infections (MOI > 1) are challenging. Single Nucleotide Polymorphisms (SNP) in the *Plasmodium chloroquine resistance transporter* (*Pfcrt*) and *Plasmodium multi drug resistance 1* (*Pfmdr-1*) have been suspected to be associated with resistance to ACT partner drugs, lumefantrine and amodiaquine [21, 22], but robust molecular markers have not yet been validated [23].

Sulfadoxine/pyrimethamine (SP) is the recommended drug for intermittent preventive treatment of pregnant women (IPTp) living in areas of moderate to high malaria transmission in Africa to prevent the deleterious effects of malaria on maternal and fetal outcomes [24]. Accumulation of point mutations at several codons in the dihydrofolate reductase (*Pfdhfr*) and dihydropteroate synthase (*Pfdhps*) genes increases the risk of SP treatment failure [25]. A quintuple mutant (51I, 59R and 108 N in the *Pfdhfr* and 437G + 540E in the *Pfdhps* genes) is a significant predictor of SP treatment failure [26, 27]. Acquisition of an additional mutation in *Pfdhps* (A581G) on the genetic background of the quintuple mutant has been shown to confer a higher degree of resistance to SP [28] and is associated with reduced efficacy of IPT-SP in pregnant women [29, 30] and in infants [31] when its frequency is above 10%.

In Liberia, malaria transmission is perennial with an estimated 1,809,994 cases and 2,232 deaths in 2019 [1, 3]. Children and pregnant women are the most affected groups. Based on a recent health facility survey, it was estimated that 33.9% of all outpatient attendance, 47.6% of admissions and 22.6% of inpatient deaths were due to malaria [32]. The Malaria Indicator Survey 2016 showed that 45% of children aged 6–59 months tested positive for malaria by rapid diagnostic test [33]. ASAQ was recommended as first-line and AL as alternative first-line (for patients who cannot tolerate ASAQ) treatments for the management of uncomplicated *P. falciparum* malaria [34]. Currently, the National Malaria Control Programme (NMCP) has reduced procurement of ASAQ and replaced it with AL as the first-line drug of choice for treatment of uncomplicated malaria and the treatment guideline will be updated (NMCP, pers. commun.). The last TES conducted in 2008–2009 showed high PCR corrected cure rates of above 97% at day 28 for both combinations [35]. Since then, the NMCP has not been able to monitor the efficacy of the recommended artemisinin-based combination due to operational challenges, including the Ebola outbreak in 2014–2015. In addition to case management and vector control, IPTp with SP is a critical component of nationally recommended interventions to reduce the burden of malaria in pregnant women [34]. To provide up to date evidence for national malaria treatment policy, the clinical and parasitological efficacy of ASAQ and AL were assessed as well as the frequency of SNP (*Pfkelch13, Pfcrt, Pfmdr-1, Pfdhfr, Pfdhps)* and CNV (*Pfpm2)* associated or suspected to be associated with resistances to anti-malarial drugs.

## Methods

### Study sites and study design

**Fig. 1:**
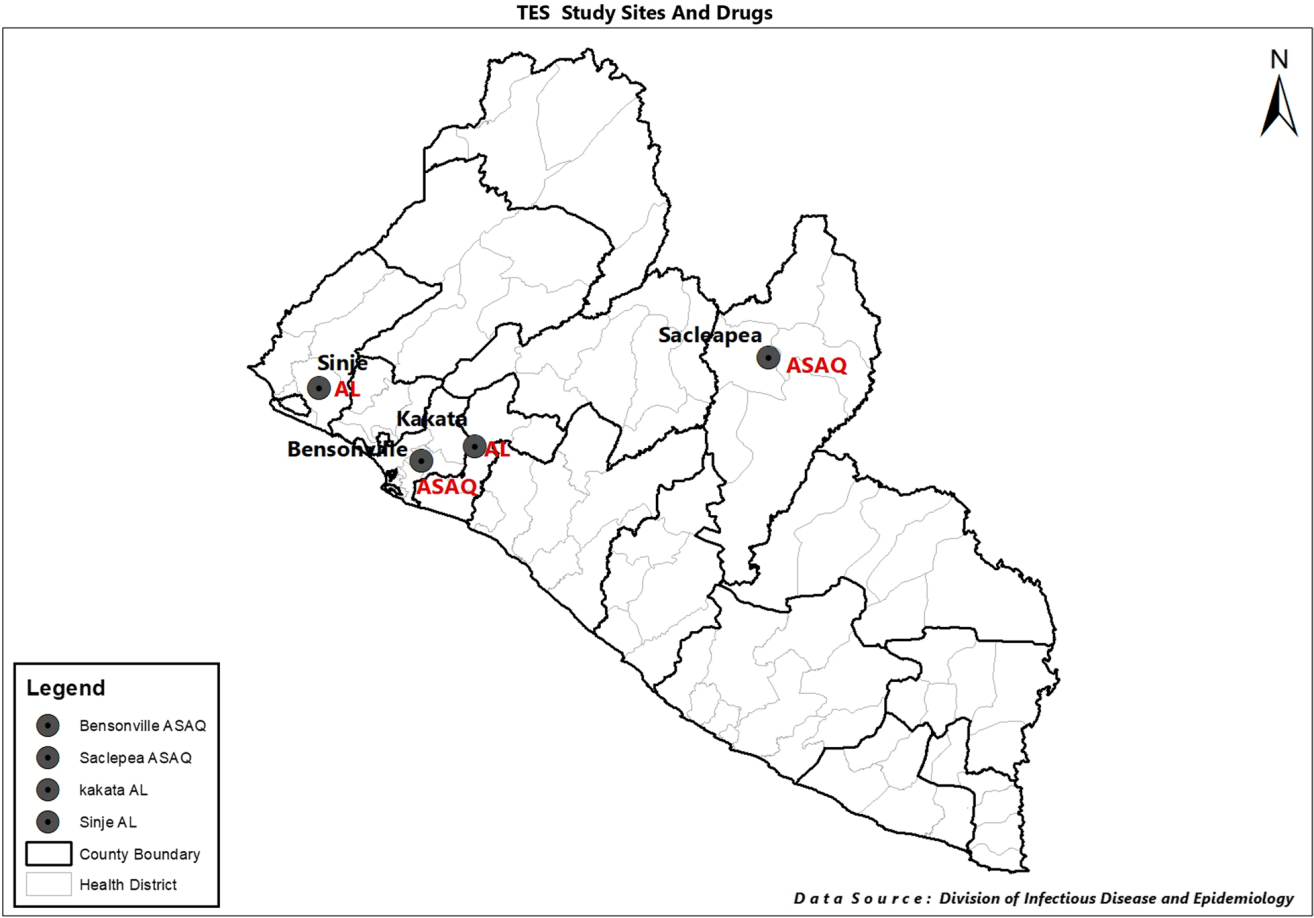
Map of Liberia showing the study sites

Study patients were recruited from four health facilities in four counties: (i) Bensonville Hospital, Bensonville, Montserrado County; (ii) Saclepea Comprehensive Health Center, Saclepea, Nimba County; (iii) Charles Henry Rennie Hospital, Kakata, Margibi County; and (iv) Sinje Health Center, Garwula, Grand Cape Mount County (Fig. 1). The efficacy of ASAQ was assessed at the Bensonville and Saclepea sites while the efficacy of AL was evaluated at the Kakata and Sinje sites. The study was one-arm cohorts evaluating the efficacy of both artemisinin-based combinations in the treatment of uncomplicated falciparum infections. Children who met the inclusion and exclusion criteria of the study were enrolled, treated with ASAQ or AL on site and assessed clinically and parasitologically for 28 days according the 2009 WHO protocol [36].

### Recruitment, treatment and follow-up procedure

Potential study children who visited the study health facilities between December 2017 and May 2018 were screened for the following criteria: age 6–59 months, axillary temperature ≥ 37.5 °C and/or history of fever in the past 24 h, and *P. falciparum* mono-infection with parasitaemia of 2000 to 200,000 asexual parasites/µl by microscopy. Other inclusion criteria included willingness to comply with the study visit schedule and informed consent from parents or guardians. Children with exclusion criteria, including the presence of general danger signs or signs of severe falciparum malaria, mixed or mono-infection with non-falciparum species, or severe malnutrition and febrile conditions due to diseases other than malaria, received appropriate care and treatment according to national guidelines.

Children recruited at the Bensonville and Saclepea sites received daily dose of ASAQ for 3 days according to the recommended weight bands: one tablet of 25 artesunate + 67.5 amodiaquine for 4.5 to < 9 kg body weight, one tablet of 50 artesunate + 135 amodiaquine for 9– < 18 kg body weight, and one tablet of 100 artesunate + 270 amodiaquine for 18 to < 36 kg body weight. A dose of artesunate 4 (range 2–10) mg/kg + amodiaquine 10 (range 7.5–15) mg/kg body weight once daily for 3 days was the target. Children from Kakata and Sinje were given twice daily dose of AL for 3 days according to recommended weight bands: one tablet for those weighing 5–14 kg and two tablets for 15–24 kg. AL was given with milk or fatty meal. All treatments were administered under direct observation by the study team and patients were observed for 30 min. If the first dose was vomited, the treatment was administered again. If vomiting recurred, the patient was given artesunate injection according to national guidelines and the patient was withdrawn from the study. Patient with treatment failure were treated with quinine 10 mg/kg BW three times a day for seven days. Prequalified ASAQ (manufactured by Sanofi with Batch numbers 5MA082 for 25 mg artesunate/67.5 mg amodiaquine and 6MA113 for 50 mg artesunate/135 mg amodiaquine, and AL (manufactured by Ipca Laboratories LTD with batch number DYI2036341) were obtained from WHO /HQ.

Study children were followed for up to 28 days at scheduled visits on days 1, 2, 3, 7, 14, 21, and 28, and at unscheduled visits when symptoms worsened or recurred. The allowable time window for the weekly follow-up was ± 1 day. A clinical assessment and parasitological examination were performed at each visit. Existing general Community Health Volunteers (gCHVs) in the study areas were engaged and trained to trace patients if they did not show up for the scheduled appointment.

### Laboratory investigation

Thick and thin blood smears collected on the day of recruitment (day 0) and follow-up days were stained with Giemsa and asexual parasites were counted against White Blood Cells (WBC) in the thick blood smear using the WHO procedure [36]. Assuming WBC count of 6,000 WBCs/μL, parasite density (asexual parasites per μL of blood) was calculated by dividing the number of asexual parasites counted by the number of WBCs and then multiplying by 6000. Two microscopists independently read all blood slides. A third microscopist re-examined the blood slides with discordant results (species diagnosis, parasite density of > 50%, or presence of parasites). The final parasite density was calculated by taking the average of the two closest counts. A blood smear was declared negative if no asexual parasites were observed after examination of 1,000 WBCs.

Filter paper blood samples were collected from each patient on day 0 and on the day of parasite recurrence (from day 7 onward), stored in individual plastic bags with desiccant and protected from light, moisture and extreme temperature until analysis. Each dried blood spot was cut out sterilely and placed in an Eppendorf tube. DNA was extracted using the QIAamp DNA Blood Mini Kit (Qiagen) as previously described [12]. Paired DNA from patients with recurrent parasites (day-0 and day of recurrence) were genotyped using nested polymerase chain reaction (PCR) targeting the highly polymorphic genes *msp1, msp2* and *glurp* [37]. The fragment sizes were estimated by capillary electrophoresis (Fragment analyzer, Agilent) and the cut-off settings for PCR artefacts and stutter peaks was defined for peaks < 10% of the low and upper control bands. The bins used to define a match were ± 10 bp for *msp1/msp2* and ± 20 bp for *glurp*. The genotypes of parasites on day 0 and on the day of parasite recurrence were compared to distinguish recrudescence (same genotype) from new infection (different genotype). Both the current WHO-recommended algorithm [38] and the newly proposed two out of three (2/3) algorithm [39] were used. In the WHO algorithm, recurrent parasitaemia was classified as recrudescence if at least one allele at all 3 markers (3/3) was common to both paired samples. In the 2/3 algorithm, recurrent parasitaemia was classified recrudescence, if at least one allele at 2 among 3 markers (2/3) was common to both paired samples.

Day 0 DNA was also analysed for the presence of point mutations in the *Pfkelch13* gene associated with artemisinin resistance [11], the *Pfcrt* and *Pfmdr-1* genes associated or suspected to be associated with 4-aminoquinolines and aminoalcohol resistance [23], and the *Pfdhfr* and *Pfdhps* genes linked to pyrimethamine and sulfadoxine resistance. Amplicons from targeted sequences were generated using nested PCR assays as previously described [40, 41] and sent to Eurofins (Germany) for sequencing. Mutations at codons 440–680 for *Pfkelch13*, at codons 72–76, 93, 97, 145, 218, 343, 350 and 353 for *Pfcrt*, at codons 86, 184, 1034, 1042 and 1246 for *Pfmdr-1*, at codons 51, 59, 108, 164 for *Pfdhfr* and at codons 436, 437, 540, 581, 613 for *Pfdhps* were assessed with the CLC Main Workbench 20 software (Qiagen). Electropherograms with mixed alleles were considered as mutant for the purpose of mutation frequency estimation. Quality control was assessed by including blinded quality control samples (parasites with wild-type, C580Y and R539T alleles for *Pfkelch13* and 3D7, Dd2, 7G8 laboratory strains for *Pfdhfr, Pfdhps, Pfcrt* and *Pfmdr-1*) in each 96-well sequencing plate. DNA from D0 samples were also analysed to estimate copy number variations in the *Pfpm2* gene, which is associated with piperaquine resistance, using the method described previously [15]. *Pfpm2* gene copy number was estimated samples without assessing the number of clones (MOI) and a *Pfpm2* copy number > 1.5 was defined as an amplification of the gene. All D0 samples were screened for *Pfkelch13* and *Pfdhfr/Pfdhps* mutations, while a *s*ubsample of 30% from each site was analysed for *Pfcrt* and *Pfmdr-1* mutations and *Pfpm2* copy number variations*.* The samples for genotyping and molecular markers were analysed at the Institut Pasteur, Paris, France.

### Outcome measures

Treatment response was classified based on parasitological and clinical response as recommended by WHO [36]: early treatment failure (ETF), late clinical failure (LCF), late parasitological failure (LPF) and adequate clinical and parasitological response (ACPR). The primary endpoint of the study was 28-day PCR-corrected ACPR. Secondary endpoints included day 3 positivity, 28-day PCR-uncorrected ACPR, and frequencies of SNP in *Pfkelch13*, *Pfdhfr*, *Pfdhps*, *Pfcrt*, *Pfmdr-1*, and CNV in *Pfmp2* genes.

### Ethical considerations

Ethical approval for the study protocol was obtained from the University of Liberia-Pacific Institute for Research & Evaluation Institutional Review Board (UL-PIRE IRB) and the WHO Research Ethics Review Committee (ERC.0002892). Parents/guardians were informed of the study procedure, its benefits and potential risks and gave written informed consent for their children to participate in the study prior to enrollment.

### Sample size and data management

A minimum sample of 73 children per site was estimated based on a 5% treatment failure rate for ASAQ and AL and with a 95% confidence level and 5% precision. Twenty percent (n = 15) was added to account for loss to follow-up and withdrawal during the 28-day follow-up period. The final target sample was 88 per site. Data were double-entered, validated against the case sheet in case of discrepancies, and analysed using the WHO excel software programme (http://www.who.int/malaria/publications/atoz/9789241597531/en/). Per-protocol and Kaplan–Meier analyses were used to analyse treatment outcomes according to the WHO protocol [36]. The per-protocol analysis was performed excluding patients who discontinued treatment, stopped treatment, withdrawn, or had new infections during follow-up. In the KM analysis, these cases were censored on the last day of follow-up, withdrawal, or re-infection. Recurrent cases with indeterminate PCR results were excluded from both the per-protocol and KM analyses. Descriptive statistics including percentages, mean, standard deviation, and range were presented. Patient characteristics at the time of enrollment and treatment outcomes were compared between sites within each study drug. Chi-square and Fisher exact tests were used to compare categorical data and t-tests were used to compare continuous variables. The presence of *Pfcrt* K76T, *Pfmdr1* N86Y andY184F mutations was compared between day 0 samples from cured and recrudescent patients using Fisher's exact test and an estimation of relative risk. A difference is considered significant if the p-value < is 0.05.

## Results

### Baseline characteristics of enrolled patients

The study was conducted from December 2017 to May 2018. A total of 359 children, 180 (91 in Bensonville and 89 Saclepea) and 179 (89 in Kakata and 90 in Sinje) were recruited for the ASAQ and AL clinical efficacy studies, respectively. Baseline characteristics of the recruited children were comparable between the sites except for mean parasite density (Table 1). Geometric mean parasite density in Sinje was significantly lower than in Saclepea (t = 3.9; df = 177; p = 0.0001) and Bensonville (t = 3.8; df = 177; p = 0.0002). In addition, lower parasite density was observed in Kakata compared to Saclepea (t = 3.0; df = 169; p = 0.003) and Bensonville (t = 3.0; df = 178; p = 0.003).

**Table 1:** Characteristics of study children at enrolment

Characteristic	Artesunate–amodiaquine		Artemether–lumefantrine	
	Bensonville (n = 91)	Saclepea (n = 89)	Kakata (n = 89)	Sinje (n = 90)
Male, n (%)	52 (57.14)	43 (48.86)	45 (50.56)	41 (45.56)
Female, n (%)	39 (42.86)	46 (51.14)	44 (49.44)	49 (54.44)
Age (years): Mean (SD^a^)	2.6 (1.2)	2.5 (1.2)	2.6 (1.3)	2.1 (1.1)
Axillary temp Mean (SD^a^)	37.1 (0.9)	38 (0.7)	37.5 (1.0)	37.4 (0.9)
Parasite density (per µL):				
Geometric mean	28,592	27,625	16,425^b^	14714^c^
CI 95%	11,781–18,375	22,303–34,217	12,625–21,369	14,712–14,714

^a^SD: standard deviation

^b^Parasiatemia in Kakata was significantly lower than that of Bensonville t = 3.8; df = 177; p=0.0002) and Saclepea (t = 3.9; df = 177; P = 0.0001)

^c^Parasite density in Sinje was significantly lower than that of Bensonville (t = 3.0; df = 178; p = 0.003) and Saclepea (t = 3.0; df = 169; p = 0.003)

### Treatment outcomes

Of the 359 children enrolled, 332 (92.5%) reached the study endpoints. Of the remaining 27 cases, 24 (17 in Bensonville, 5 in Kakata and 2 in Sinje) were lost to follow-up and three (2 in Bensonville and 1 in Saclepea) were withdrawn during follow-up due to missing treatment dose for day-1 (one case) or for day-2 (two cases). Before PCR correction, per-protocol analysis revealed an ACPR of 63.9% (51.7–74.9%) and 86.4% (77.4–92.8%) for ASAQ treatment in Bensonville and Saclepea, respectively, while ACPR of 94.0% (86.7–98.0%) and 100% (95.9–100%) were observed for AL in Kakata and Sinje, respectively (Table 2). Forty-three patients experienced parasite recurrences, most of which occurred in the ASAQ group in Bensonville (26/43, 60.5% recurrences) and Saclepea (12/43, 27.9% recurrences). Most of these parasite recurrences were new infections (31/43, 74.1%), of which the majority (20/31, 64.5%) occurred in Bensonville.

**Table 2:** 28-day PCR-unadjusted treatment efficacy of study children after treatment with Artesunate–amodiaquine or Artemether–lumefantrine

*PCR-unadjusted* outcome	Artesunate–amodiaquine				Artemether–lumefantrine			
	Bensonville (n = 91)		Saclepea (n = 89)		Kakata (n = 89)		Sinje (n = 90)	
	n (%)	95% CI	n (%)	95% CI	n (%)	95% CI	n (%)	95% CI
LCF	6 (8.3)	3.1–17.3	3 (3.4)	0.7–9.6	1 (1.2)	0.0–6.5	0 (0)	0.0–4.1
LPF	20 (27.8)	17.9–39.6	9 (10.2)	4.8–18.5	4 (4.8)	1.3–11.7	0 (0)	0.0–4.1
ACPR	46 (63.9)	51.7–74.9	76 (86.4)	77.4–92.8	79 (94.0)	86.7–98.0	88 (100)	95.9–100
Total per protocol	72		88		84		88	
Withdrawn/lost	19 (20.9)		1 (1.1)		5 (5.6)		2 (2.2)	
Kaplan Meier: cure rate	65.1	53.0–74.8	86.4	77.2–92.0	94.0	86.3–97.5	100	n/a

*LCF* late clinical failure,* LPF* late parasitological failure,* ACPR* adequate clinical and parasitological response

Using the PCR analysis method recommended by the WHO [38], the PCR-corrected per protocol results showed an ACPR of 90.2% (78.6–96.7%) in Bensonville and 92.7% (84.8–97.3%) in Saclepea for ASAQ (Table 3). PCR-corrected KM analysis revealed cumulative cure rate of 92.1% (81.9–96.6%) and 93.0% (85.1–96.8%) in Bensonville and Saclepea, respectively. For AL, both the per-protocol and KM analysis showed 100% efficacy at both sites. Compared with the WHO algorithm, the 2/3 algorithm showed that all recurrences classified as recrudescence (n = 11) remained the same while 25.8% (8/31) of the new infections were reclassified as recrudescence (see Additional file 1: Genotyping Raw data). These changes primarily affected the Bensonville site, where 30% (6/20 of the new infections were re-assigned to recrudescence, reducing the efficacy rate from 90.2% to 80.7% (Table 4). On day2, 4.5% (4/89) of patients at Bensonville, 2.3% (2/88) at Saclepea, 17.0% (15/88) at Sinje and 4.6% (4/87) at Kakata were still slide positive. Except for two cases (0.6%, 2/352), children in both treatment groups were free of parasites on day3.

**Table 3:** 28-day PCR corrected treatment outcomes: WHO algorithm

PCR adjusted Outcome	Artesunate–amodiaquine					Artemether–lumefantrine				
	Bensonville (n = 91)		Saclepea (n = 89)		Overall (n = 180)	Kakata (N = 89)		Sinje (N = 90)		Overall (n = 179)
	n (%)	CI 95%	n (%)	95% CI	% (CI 95%)	n (%)	CI 95%	n (%)	95% CI	% (CI 95%)
LCF^a^	0	0.0–7.1	2 (2.4)	0.3–8.5	1.5 (0.2–5.3)	0	0.0–4.6	0 (0)	0.0–4.1	0 (0.0–2.2
LPF^b^	5 (9.8)	3.3–21.4	4 (4.9)	1.3	7.5 (3.6–13.3)	0	0.0–4.6	0 (0)	0.0–4.1	0 (0.0–2.2
ACPR^c^	46 (90.2)	78.6–96.7	76 (92.7)	84.8–97.3	91.0 (84.9–95.3)	79 (100)	95.4–100	88 (100)	95.9–100	100 (97.8–100)
Total per-protocol	51		82		134	79		88		167
Withdrawn/lost:	19 (20.9)		1 (1.1)		20 (11.1)	5 (5.6)		2 (2.2)		7 (3.9)
Re-infection	20 (23.1)		5 (5.6)		25 (13.9)	5 (5.6)		0		5
Unknown	1 (1.1)		0		1 (0.6)	0		0		0
KM^d^ cure rate	92.1	81.9–96.6	93.0	85.1–96.8	92.0 (86.4–95.4)	100	NA	100	NA	100 (NA)

^a^* LCF* late clinical failure,* bLPF* late parasitological failure,* cACPR* adequate clinical and parasitological response,* dKM* Kaplan Meier

**Table 4:** 28-day PCR corrected treatment outcome: 2/3 algorithm

PCR adjusted Outcome	Artesunate–amodiaquine					Aretemether-lumefantrine				
	Bensonville (n = 91)		Saclepea (n = 89)		Overall (n = 180)	Kakata (N = 89)		Sinje (N = 90)		Overall (N = 179)
	n (%)	CI 95%	n (%)	95% CI	% (CI 95%)	n (%)	95% CI	n (%)	95% CI	% (CI 95%)
LCF^a^	1 (1.8)	0.0–9.4	2 (2.4)	0.3–8.4	2.1 (0.4–6.1	0	0.0–4.5	0 (0)	0.0–4.1	0 (0.0–2.2)
LPF^b^	10 (17.5)	8.7–29.9	5 (6.0)	2.0–13.5	10.7 (6.1–17.1)	1 (1.3)	0.0–6.8	0 (0)	0.0–4.1	0.6 (0.0–3.3)
ACPR^c^	46 (80.7)	68.1–90.0	76 (91.6)	83.4–96.5	87.1 (80.4–92.2)	79 (98.8)	93.2–100	88 (100)	95.9–100	99.4 (96.7–100)
Total per-protocol	57		83		140	80		88		168
Withdrawn/lost:	19 (20.9)		1 (1.1)		20 (11.1)	5 (5.6)		2 (2.2)		7 (3.9)
Re-infection	13		5		18	4		0		4
PCR NI^d^	2		0		2	0			0	0
^e^KM cure rate	84.1	73.0–90.9	91.8	83.6–96.0	88.4 (82.2–92.5)	98.8		100		99.4 (95.9–99.9)

^a^* LCF* late clinical failure,* bLPF* late parasitological failure,* cACPR* adequate clinical and parasitological response, *eKM* Kaplan Meier

### Markers for partial artemisinin resistance

Of the 359 pretreatment samples, 358 (99.7%) yielded interpretable results, most of which (353/358) carried the *Pfkelch13* wild-type allele, ranging from 95.6 to 100% depending on the site. The remaining five isolates carried synonymous mutants (C469C in one sample at Bensonville, G548G in two samples at Bensonville and Kakata, and Y493Y in one sample at Bensonville) and one non-synonymous mutant (V637I in one sample at Saclepea).

### Markers for piperaquine resistance

Estimation of *Pfpm2* copy number was performed on 112 randomly selected day-0 samples (25% of day-0 samples per site). Interpretable data were obtained for 100 samples (89.3%). Samples that were not interpretable were likely samples with insufficient DNA quantity (due to initial parasite density) or poor DNA quality. Parasites in all samples carried a single copy of the *Pfpm2* gene (CNV < 1.5).

### Associations between mutations in the Pfcrt and Pfmdr-1 genes and ASAQ or AL clinical and parasitological outcomes

A total of 91 day 0 samples from patients treated with AL or ASAQ were selected and used to assess the frequency of mutations in the *Pfcrt* and *Pfmdr-1* genes. These samples included all the day 0 isolates from patients classified as recurrent (n = 40, 5 in the AL group and 35 in the ASAQ group) and a random selection of day-0 samples from patients classified as cured (n = 43, 28 in the AL group and 15 in the ASAQ group) or lost to follow-up (n = 8, 3 in the AL group and 5 in the ASAQ group).

Molecular analysis revealed that the *Pfcrt* 76 T mutant was highly frequent (87.9%, 80/91), with little variation in frequencies between sites (80.0% to 95.2%) (Table 5). The 74I/75E/76 T/356 T (44.0%,40/91), followed by 74I/75E/76 T (31.9%, 29/91) and the wild-type allele (12.1%, 11/91) were the most frequent *Pfcrt* alleles. For the *Pfmdr-1* gene, the 184F mutation was the most common mutation (51.6%, 47/91), followed by the 86Y mutation (44.0%, 40/91). Across the study sites, the proportions of the two mutants were not much different (Table 5), except in Sinje site, where the frequencies of 184F and 86Y were different (66.5% and 26.7%, respectively). Next, the association of *Pfcrt* K76T, *Pfmdr-1* N86Y, and Y184F mutations with adjusted 28-day clinical outcomes were examined. As shown in Table 6, no association between the presence of the mutations in *Pfcrt* and *Pfmdr-1* and the risk of parasite recrudescence in patients treated with ASAQ (defined by the WHO and the 2/3 algorithms) was observed. *Pfmdr-1* N86 and 86Y were equally distributed in the AL group and none of the genotype was associated with treatment failure.

**Table 5:** *Pfcrt* and *Pfmdr-1* mutation and alleles observed in *P. falciparum* obtained from blood samples collected prior antimalarial treatment

		Bensonville		Saclepea		Kakata		Sinje		Total	
		n	%	n	%	n	%	n	%	N	%
Mutation	*Pfcrt*										
	76 T	29	85.3	19	90.5	20	95.2	12	80.0	80	87.9
	*Pfmdr-1*										
	86Y	16	47.1	8	38.1	12	57.1	4	26.7	40	44.0
	184F	16	47.1	10	47.6	11	52.4	10	66.7	47	51.6
Alleles	*Pfcrt*										
	WT	5	14.7	2	9.5	1	4.8	3	20.0	11	12.1
	74I/75E/76 T	11	32.4	8	38.1	6	28.6	4	26.7	29	31.9
	74I/75E/76 T/362 V	0	0.0	0	0.0	0	0.0	1	6.7	1	1.1
	74I/75E/76 T/356 T	16	47.1	8	38.1	10	47.6	6	40.0	40	44.0
	74I/75E/76 T/217F/356 T	0	0.0	1	4.8	0	0.0	0	0.0	1	1.1
	74I/75E/76 T/141L	0	0.0	1	4.8	2	9.5	0	0.0	3	3.3
	74I/75E/76 T/141L/356 T	2	5.9	1	4.8	2	9.5	1	6.7	6	6.6
	Total	34	100.00	21	100.00	21	100.00	15	100.00	91	100.00
	*Pfmdr-1*										
	WT	9	26.5	5	23.8	5	23.8	2	13.3	21	23.1
	86Y	6	17.6	5	23.8	5	23.8	1	6.7	17	18.7
	184F	9	26.5	8	38.1	4	19.0	9	60.0	30	33.0
	86Y/1246Y	3	8.8	1	4.8	0	0.0	2	13.3	6	6.6
	86Y/184F	6	17.6	2	9.5	7	33.3	1	6.7	16	17.6
	86Y/184F/1276Y	1	2.9	0	0.0	0	0.0	0	0.0	1	1.1
	Total	34	100.00	21	100.00	21	100.00	15	100.00	91	100.00

**Table 6:** Associations between *Pfcrt K76T, Pfmdr-1 N86Y* and Y*184F* mutations with unadjusted (recurrences) and adjusted (recrudescences) 28-day clinical outcomes following ASAQ treatment

Outcome	Algorithm decision	Parameters	Mutation					
			*Pfcrt*		*Pfmdr-1*			
			K76	76 T	N86	86Y	Y184	184F
Unadjusted outcome		Cured	1	18	4	15	6	13
		Recurrence	6	30	10	26	23	13
		Mean survival (SE), days	23.0 (2.5)	25.2 (0.6)	24.5 (1.4)	25.1 (0.7)	24.8 (0.9)	25.0 (0.9)
		Logrank test, *p* value	0.18		0.58		0.10	
		Hazard ratio (95% CI)	2.4 (0.7–8.5)		1.3 (0.5–3.2)		1.9 (0.9–4.3)	
Adjusted outcome	WHO/MMV	Cured	6	37	13	30	21	22
		Recrudescence	1	11	1	11	8	4
		Mean survival (SE), days	28.0 (0)	27.2 (0.4)	28.0 (0)	27.1 (0.5)	27.4 (0.4)	27.2 (0.7)
		Logrank test, *p* value	0.76		0.16		0.31	
		Hazard ratio (95% CI)	0.7 (0.1–5.3)		0.4 (0.08–1.5)		1.8 (0.5–6.4)	
	2/3	Cured	5	31	11	25	19	17
		Recrudescence	1	17	2	16	9	9
		Mean survival (SE), days	28.0 (0)	26.5 (0.5)	26.9 (1.4)	26.5 (0.5)	26.9 (0.6)	26.3 (0.7)
		Logrank test, *p* value	0.44		0.15		0.79	
		Hazard ratio (95% CI)	0.6 (0.1–2.7)		0.4 (0.1–1.3)		0.8 (0.3–2.4)	

### Markers for SP resistance

The *Pfdhfr* and *Pfdhps* genes were successfully amplified in 99.7% (358/359) and 99.4% (357/359), respectively. For the *Pfdhfr* gene, the triple mutant (51I/59R/108 N) was the predominant allele and accounted for 49.2%, with little variation between sites (Table 7). Other alleles detected included the 108 N allele (36.3%) and the wild-type allele (14.5%). For the *Pfdhps* gene, the wild-type allele was the most common (47.9%), followed by the 540E allele (16.0%), the 436A allele (14.3%), and the 436A/437G double mutant allele (9.0%). No significant differences were observed between sites. The 581G mutation, associated with loss of protective efficacy of IPTi and IPTp, was rare and observed in seven samples (7/357, 2%). The most frequent *Pfdhfr/Pfdhps* haplotypes were 51I/59R/108 N-wild type (25.2%), followed by 108 N-wild type (14.8%) and wild type-wild-type (7.8%), as described in Table 8. The frequency of the quintuple mutant haplotype (51I/59R/108 N-437G/540E) was found only in one (0.3%) isolate from Bensonville. No sextuple mutant haplotype (quintuple + 581G) was detected in the samples tested.

**Table 7:** *dhfr* and *dhps* alleles observed in *P. falciparum* obtained from blood samples collected prior antimalarial treatment

	Bensonville (n = 91)		Saclepea (n = 89)		Kakata (n = 89)			Sinje Town (n = 90)		Total (n = 359)	
	n	%	n	%	n	%	n		%	N	%
*dhfr*											
WT	14	15.6%	5	5.6%	9	10.1%	24		26.7%	52	14.5%
108 N	43	47.8%	25	28.1%	32	36.0%	30		33.3%	130	36.3%
51I/59R/108 N	33	36.7%	59	66.3%	48	53.9%	36		40.0%	176	49.2%
Total*	90	100.0%	89	100.0%	89	100.0%	90		100.0%	358	100.0%
*dhps*											
WT	26	29.2%	43	48.3%	52	58.4%	50		55.6%	171	47.9%
436A	7	7.9%	13	14.6%	12	13.5%	19		21.1%	51	14.3%
437G	15	16.9%	0	0	1	1.1%	1		1.1%	17	4.8%
K540E	15	16.9%	15	16.9%	15	16.9%	12		13.3%	57	16.0%
A613S	2	2.2%	3	3.4%	0	0	4		4.4%	9	2.5%
436A/437G	17	19.1%	8	9.0%	3	3.4%	4		4.4%	32	9.0%
436A/613S	0	0	4	4.5%	4	4.5%	0		0	8	2.2%
S436A/K540E	1	1.1%	0	0	0	0	0		0	1	0.3%
437G/581G	1	1.1%	0	0	1	1.1%	0		0	2	0.6%
437G/540E	1	1.1%	0	0	0	0	0		0	1	0.3%
436A/437G/540E	1	1.1%	0	0	0	0	0		0	1	0.3%
436A/437G/613S	2	2.2%	0	0	0	0	0		0	2	0.6%
436A/581G/613S	0	0	0	0	1	1.1%	0		0	1	0.3%
436A/437G/581G/613S	1	1.1%	3	3.4%	0	0	0		0	4	1.1%
Total **	89	100.0%	89	100.0%	89	100.0%	90		100.0%	357	100.0%

*One sample from Bensonville gave not interpretable data for dhfr sequence. ** two samples from Bensonville gave not interpretable data for dhps sequence

**Table 8:** *dhfr/dhps* haplotypes observed in *P. falciparum* obtained from blood samples collected prior antimalarial treatment

Haplotype			Bensonville (n = 91)		Saclepea (n = 89)		Kakata (n = 89)		Sinje Town (n = 90)		Total (n = 359)	
			n	%	n	%	n	%	n	%	N	%
Combined	*dhfr*	*dhps*										
Wild type	WT	WT	5	5.6%	1	1.1%	7	7.9%	15	16.7%	28	7.8%
Single mutant	WT	436A	0	0	1	1.1%	0	0	4	4.4%	5	1.4%
	WT	437G	2	2.2%	0	0	0	0	0	0	2	0.6%
	WT	613S	1	1.1%	0	0	0	0	1	1.1%	2	0.6%
	WT	K40E	3	3.4%	1	1.1%	1	1.1%	4	4.4%	9	2.5%
	108 N	WT	9	10.1%	8	9.0%	21	23.6%	15	16.7%	53	14.8%
Double mutant	WT	436A/437G	3	3.4%	2	2.2%	0	0	0	0	5	1.4%
	108 N	436A	5	5.6%	5	5.6%	4	4.5%	7	7.8%	21	5.9%
	108 N	437G	8	9.0%	0	0	0	0	1	1.1%	9	2.5%
	108 N	540E	10	11.2%	4	4.5%	5	5.6%	4	4.4%	23	6.4%
	108 N	613S	1	1.1%	2	2.2%	0	0	0	0	3	0.8%
Triple mutant	WT	S436A/A581G/A613S	0	0	0	0	1	1.1%	0	0	1	0.3%
	51I/59R/108 N	WT	12	13.5%	34	38.2%	24	27.0%	20	22.2%	90	25.2%
	108 N	436A/437G	8	9.0%	2	2.2%	0	0	3	3.3%	13	3.6%
	108 N	436A/613S	0	0	1	1.1%	1	1.1%	0	0	2	0.6%
	108 N	437G/581G	1	1.1%	0	0	1	1.1%	0	0	2	0.6%
Quadruple mutant	51I/59R/108 N	436A	2	2.2%	7	7.9%	8	9.0%	8	8.9%	25	7.0%
	51I/59R/108 N	437G	5	5.6%	0	0	1	1.1%	0	0	6	1.7%
	51I/59R/108 N	540E	2	2.2%	10	11.2%	9	10.1%	4	4.4%	25	7.0%
	51I/59R/108 N	613S	0	0	1	1.1%	0	0	3	3.3%	4	1.1%
Quintuple mutant	51I/59R/108 N	436A/437G	6	6.7%	4	4.5%	3	3.4%	1	1.1%	14	3.9%
	51I/59R/108 N	436A/540E	1	1.1%	0	0	0	0	0	0	1	0.3%
	51I/59R/108 N	436A/613S	0	0	3	3.4%	3	3.4%	0	0	6	1.7%
	**51I/59R/108 N**	**437G/540E**	1	1.1%	0	0	0	0	0	0	1	0.3%
	108 N	437G/581G/613S	0	0	0	0	3	3.4%	0	0	3	0.8%
Sextuple mutant	51I/59R/108 N	436A/437G/613S	2	2.2%	0	0	0	0	0	0	2	0.6%
Septuple mutant	**51I/59R/108 N**	**436A/437G/581G/613S**	1	1.1%	0	0	0	0	0	0	1	0.3%
Total *			89	100.0%	89	100.0%	89	100.0%	90	100.0%	357	100.0%

*Two samples from Bensonville gave not interpretable data for dhfr/dhps sequences

## Discussion

Using the WHO recommended PCR analysis [38], the current study conducted eight years after the last therapeutic efficacy study showed cure rates of 90.2% (95% CI: 78.6–96.7%) in Bensonville and 91.6% (83.4–96.5%) in Saclepea for ASAQ, suggesting a possible decline in the efficacy of this ACT compared to the status in 2009 [35]. Bensonville is the capital of Montserrado with a high population movement. The contact information provided for follow-up sometimes not reachable, or the proxy contact telephone number provided for follow-up was sometimes not close to the patient/client. These factors may have contributed to the high rate (18.7%) of lost to follow-up in this study site. The study also revealed PCR corrected AL cure rate of 100% observed in the study sites demonstrate that AL remained highly efficacious. In contrast to the results of the current study, ASAQ has maintained its high efficacy in neighbouring [42–44] and other West African countries [45–50] with cure rates of 98% and above. Similar high cure rates (96% and above) with the AL treatment have also been reported in the sub-region [42–51]. Both artemisinin-based combinations also remain highly effective in other African countries [52–61].

In the current study, parasite genotyping to distinguish between reinfection and recrudescence (true failures) was analysed using both the WHO [38] and the proposed 2/3 [39] algorithms. Compared to the WHO algorithm, the proposed 2/3 algorithm classified reinfection as recrudescence (25.8%), resulting in an increase in treatment failure rates. These changes primarily affected Bensonville, where 30% of new infections as per WHO algorithm were reclassified as recrudescence resulting in a drop of the efficacy rate to 80.7%. Based on both analysis (WHO or 2/3), the results seem to show a loss of efficacy of ASAQ in Bensonville lies between equal or below the 90% threshold at which a change in treatment policy should be initiated [3]. The results of this study support the recent decision of NMCP to replace ASAQ with AL as first-line treatment of uncomplicated falciparum malaria (NMCP, pers. commun.). The high rate of new infections in Bensonville shown by both algorithms could indicate an ongoing high malaria transmission. In such a setting, high multiplicity of infection (MOI) and high rates of new infection pose a challenge in distinguishing between recrudescence and reinfection and analysis copy numbers of markers of resistance. A similar significant difference in failure rates between the WHO and 2/3 algorithms in a high transmission area was recently reported from Equatorial Guinea [55].

In a recent WHO analysis, overall, the proportion of recurrent parasitsemia classified as recrudescence was higher with the 2/3 algorithm than with the WHO method (p < 0.001). However, this did not always translate into a significant difference in Kaplan–Meier estimates of treatment outcome. Differences in the Kaplan–Meier estimates of treatment outcome were more evident in areas of moderate to high transmission than in areas of low to moderate transmission in particular for artemether–lumefantrine. Though there is no gold standard, an expert committee recommended that WHO methodology [38] should be maintained as the primary analysis methodology for reporting and policy change. Bayesian and 2/3 algorithms may be applied for evaluation and comparison, but not for primary reporting.

In the current study, parasites were cleared by day 3 in all but two patients, which, together with the absence of the *Pfkelch13* mutation known to be associated with artemisinin resistance, indicates absence of artemisinin resistance in Liberia. Until recently, *Pfkelch13* mutations known to be associated with artemisinin resistance were rare or absent in Africa [19, 51, 55, 62–65]. However, this landscape has recently changed. For instance, indigenous *Pfkelch13* R561H mutant was detected in 7.3% of samples collected from the Masaka site in Rwanda between 2013 and 2015, but without delayed parasite clearance [12]. A subsequent study in 2018 showed a higher prevalence of the R561H mutant in Masaka (16%) and Rukara (15%), which was associated with delayed parasite clearance as measured by parasitaemia on day3 [13]. In addition, an association between *Pfkelch13* A675V or C469Y, candidate mutations, and prolonged parasite clearance half-life following artemisinin monotherapy was reported from Uganda [14]. These findings are concerning and highlight the need for frequent monitoring of ACT efficacy, including clearance of parasitaemia, and *Pfkelch13* mutations in Africa, as recommended by the WHO [3].

Dihydroartemisinin–piperaquine has recently been adopted as a second-line treatment for uncomplicated malaria and as a drug for Mass Drug Administration (MDA) in Africa. No parasites with multiple copies of the *Pfpm2* gene were detected in the current study. CNV data, however, from polyclonal infections are not completely reliable. Indeed, it remains a challenge to assess copy number variations in multi-genome infections, as minor variants with amplified plasmepsin 2–3 maybe be missed. A recent study found a high frequency of multiple copies of the *Pfpm2* gene in African samples, varying from 11 to 34% [20]. Given the experience in Southeast Asia, where piperaquine resistance has emerged and spread rapidly, resulting in high treatment failure rates after DP treatment, *Pfpm2* gene amplification should be closely monitored in Africa.

A medium to high frequency of parasites carrying polymorphisms in the *Pfcrt* and *Pfmdr-1* genes was observed, which could be explained by the predominant use of ASAQ in the country. However, no association between the presence of the mutations in *Pfcrt* and *Pfmdr-1* and the risk of parasite recrudescence in patients treated with ASAQ was observed. Overall, this clearly points out that robust molecular markers associated with amodiaquine and lumefantrine are still lacking.

Intermittent preventive treatment of malaria in pregnancy with SP (IPTp- SP) is one of the recommended core interventions in areas of moderate to high malaria transmission in Africa, including Liberia [24, 34]. As the effectiveness of this strategy is threatened by SP resistance, monitoring of mutations in the *Pfdhfr* and *Pfdhps* genes is an important tool to determine the status of SP resistance and guide IPTp policy. Although quintuple *Pfdhfr/Pfhps* mutations (N51I/C59R/S108N-A437G/K540E) have been associated with clinical SP treatment failure [26, 27], evidence suggests that IPTp-SP remains effective in areas with high prevalence of quintuple mutation [24]. However, reduced effectiveness of IPT-SP has been reported in infants and pregnant women in areas where parasites with sextuple mutation (quintuple + 581G) are present [66, 67]. In the current study, the *Pfdhfr* triple mutation (N51I/C59R/S108N) was the most common *Pfdhfr* allele detected, accounting for 49.2%. The very low frequency (1/357, 0.3%) of the quintuple mutant haplotype and the absence of the sextuple mutation (quintuple-581G) support the continued use of SP for IPTp in Liberia. Due to the high proportion of polyclonal infections detected by *msp1/msp2/glurp* genotyping, we cannot infer *Pfdhfr/Pfdhps* haplotypes with absolute certainty because the combination of SNPs could be deduced from different clones. The prevalence of *Pfdhfr* and *Pfdhps* mutations varies across the continent from absent to a low prevalence of quintuple mutations in West Africa [68–71] and a very high prevalence (> 70%) in East Africa [72–76]. As expected, mutations in *Pfdhfr* and *Pfdhps* genes will continue to evolve to saturation under SP drug pressure in moderate to high transmission settings in Africa, where IPT with SP is recommended [77]. Therefore, it is important to continuously monitor the markers for SP resistance.

## Conclusion

The findings of this study report a decline in the efficacy of ASAQ, while AL remains highly effective, supporting the recent decision by NMCP to replace ASAQ with AL as first-line treatment for uncomplicated falciparum malaria. There are no parasites carrying signatures known to be associated with artemisinin and piperaquine resistance. No association between the presence of the mutations in *Pfcrt* and *Pfmdr-1* and the risk of parasite recrudescence in patients treated with ASAQ was observed. The very low frequency of the *Pfdhfr/Pfdhps* five-mutant haplotype supports the continued use of SP as an IPTp. The therapeutic efficacy of recommended artemisinin-based combination, molecular markers of resistance to artemisinin, partner drugs and SP should be closely monitored for early detection of resistant parasites and development of evidence-based malaria treatment and chemoprevention strategies.

## Supplementary Information

**Additional file 1.** Liberia genotyping raw data.

## Data Availability

The dataset used in this study is available and can be shared upon reasonable request to NMCP through the corresponding author.

## Abbreviations

ACPR: Adequate clinical and parasitological response
AL: Artemether–lumefantrine
ASAQ: Artesunate–amodiaquine
ASMQ: Artesunate–mefloquine
ASPY: Artesunate–pyronaridine
ASSP: Artesunate–sulfadoxine/pyrimethamine
DP: Dihydroartemisinin–piperaquine
ETF: Early treatment failure
gCHVs: General Community Health Volunteers
glurp: Glutamate-rich protein
IPTp: Intermittent preventive treatment of pregnant women
LCF: Late clinical failure
LPF: Late parasitological failure
MDA: Mass Drug Administration
*Pfcrt*: Plasmodium falciparum chloroquine resistance transporter
*Pfmdr-1*: Plasmodium falciparum multi drug resistance 1
*Pfkelch13*: *Plasmodium falciparum* Kelch13
*Pfpm2*: Plasmepsin‑2
*Pfdhfr*: Plasmodium falciparum dihydrofolate reductase-thymidylate synthase
*Pfdhps*: Plasmodium falciparum dihydropteroate synthetase
HQ: Headquarters
MOH: Ministry of Health
msp1: Merozoite surface proteins 1
msp2: Merozoite surface proteins 2
NMCP: National Malaria Control Programme
PCR: Polymerase chain reaction
WBC: White Blood Cell
WHO: World Health Organization
